# Secretory products from regulatory macrophages modulate senescence in human endothelial cells: implications for cardiovascular aging and diseases

**DOI:** 10.1186/s12872-026-05732-w

**Published:** 2026-03-31

**Authors:** Katharina Delfs, Rouven Berndt, Katharina Hess, Christine Eimer, Fred Fändrich, Markus Steinfath, Karina Zitta, Martin Albrecht

**Affiliations:** 1 Department of Anesthesiology and Intensive Care Medicine, University Hospital Schleswig-Holstein, Kiel, Germany; 2 Heart and Vascular Center, University Medical Center Hamburg-Eppendorf, Hamburg, Germany; 3 Institute for Clinical Research and Systems Medicine, Health and Medical University Potsdam, Potsdam, Germany; 4 Department for Applied Cell Therapy, University Hospital Schleswig–Holstein, Kiel, Germany

**Keywords:** Cardiovascular diseases, Endothelial cells, Regulatory macrophages, Senescence

## Abstract

**Background:**

Cardiovascular diseases (CVDs) are the leading cause of mortality worldwide and primarily affect older individuals, making CVDs a central concern in age-related and geriatric medicine. Senescent endothelial cells contribute to a pro-inflammatory environment that exacerbates cardiovascular pathology. Therapies targeting senescent cells, hold promise for treating/preventing CVDs and anti-inflammatory regulatory macrophages (Mreg) may represent a novel cell-based therapeutic approach. Here we investigated the impact of Mreg secretory products (SP_Mreg_) on the early and late passage phenotype of human endothelial cells (HUVEC, human umbilical vein endothelial cells), with implications for cardiovascular aging and diseases.

**Methods:**

HUVEC were classified as early passage (HUVEC_ep_, passages 4–6) or late passage (HUVEC_lp_, passages 10–13) and cultured for 10 days with or without SP_Mreg_. Parameters associated with endothelial aging, including cell morphology, size and volume, β-galactosidase activity, CD105 expression, reactive oxygen species (ROS), and senescence-associated secretory phenotype (SASP) factor release, were analyzed.

**Results:**

HUVEC_ep_ exhibited a typical cobblestone-like morphology, whereas HUVEC_lp_ displayed a spindle-shaped appearance. Both cell types showed an elongated, fibroblast-like cell type after incubation with SP_Mreg_. In HUVEC_lp_, treatment with SP_Mreg_ significantly reduced cell size and volume at all time points (*P* < 0.05). In HUVEC_ep_, SP_Mreg_ increased β-galactosidase activity and the proportion of ROS-positive cells, while reducing secretion of the SASP factor PAI-1 (*P* < 0.05 for all). In HUVEC_lp_ SP_Mreg_ increased β-galactosidase activity, attenuated the time-dependent increase of ROS levels and secretion of SASP factor Activin A (*P* < 0.05 for all).

**Conclusion:**

Our findings demonstrate that SP_Mreg_ modulate several factors associated with endothelial senescence, highlighting a potential role for Mreg in processes related to cardiovascular aging.

**Supplementary Information:**

The online version contains supplementary material available at 10.1186/s12872-026-05732-w.

## Introduction

Cardiovascular diseases (CVDs) are the main cause of morbidity and mortality worldwide and represent a heterogeneous group of chronic diseases affecting heart and blood vessels [1]. Due to demographic changes, the prevention and treatment of CVDs, as well as the development of novel therapeutic strategies, have gained increasing attention over recent decades [2, 3].

Cardiovascular aging and premature senescence of endothelial cells play a central role in the development of CVDs [2, 4]. Endothelial cells forming the inner lining of blood vessels constitute the first barrier between the blood stream and the surrounding tissue. Under physiological conditions, endothelial cells contribute to the prevention of plaque formation and vasoconstriction [5]. When endothelial cells become senescent upregulation of adhesion molecules, increased vascular permeability and vasoconstriction can be the consequences. These effects are caused among other things by the induction of a chronic inflammation promoting the CVD development in aged individuals [6, 7].

Targeting cardiovascular aging (i.e. senescent endothelial cells) could be a potential therapeutic approach in preventing and treating CVDs. Cellular senescence is defined as stable, irreversible cell cycle arrest caused by different stimuli such as telomere shortening, oxidative stress, mitochondrial damage or radiation [8]. Senescence can have positive and negative effects on the surrounding tissue, referred to as the pleiotropy of senescence [9]. Firstly, senescence is a potential tumor suppressing mechanism by limiting the proliferation of damaged cells, has important roles during the physiological development and in the regulation of the tissue homeostasis [10, 11]. But the accumulation of senescent cells can also induce a proinflammatory environment which can cause a chronic sterile inflammation resulting in the development of many age-related diseases like atherosclerosis, Alzheimer disease or diabetes mellitus [12, 13]. Because of the lack of one specific senescence marker a combination of senescence markers selected specifically for cell type and senescence induction method must be applied to identify senescent cells *in-vitro* and *in-vivo.* Commonly used markers for the identification of senescent endothelial cells are β-galactosidase, CD105, reactive oxygen species (ROS) and specific senescence associated secretory phenotype (SASP) factors [13, 14].

Identifying senescent cells and understanding their specific markers is essential for developing targeted therapeutic approaches. Building on these insights, innovative strategies such as cell-based therapeutics may represent promising avenues for the treatment and prevention of CVDs [3, 15]. In the last years regulatory macrophages (Mreg) gained attention by the identification of their immunosuppressive and anti-inflammatory effects as well as their positive effects on angiogenesis [16, 17]. Mreg are originated from peripheral blood monocytes and can be generated by incubation with M-CSF, human serum and an additional short-term stimulation with IFN-gamma *in-vitro* [18]. It has been shown that high levels of Mreg in blood vessels reduce the development of atherosclerotic lesions by increasing IL-10 production [19]. Whether Mreg also play a role in the context of cardiovascular aging and senescence-associated processes is currently unknown.

In the present study, we show that secretory products from regulatory macrophages modulate several senescence-related parameters in human endothelial cells, suggesting a possible therapeutic potential of Mreg in cardiovascular aging and associated diseases.

## Methods

### Isolation and cultivation of HUVEC (human umbilical vein endothelial cells)

The study was approved by the local Ethics Committee of the University Medical Center Schleswig–Holstein, Kiel, Germany (protocol identification: D519/18 and D518/13). In this study a HUVEC based cell culture model was employed. HUVEC are of fetal origin and derived from venous tissue. Although they do not fully reflect the phenotype and biology of adult arterial endothelial cells, HUVEC are among the best-characterized primary endothelial cell types and offer high reproducibility, broad availability, and well-defined stress and activation responses. These features make them a widely accepted model for mechanistic in vitro studies, despite their inherent developmental and vascular-bed–specific limitations [20]. HUVEC were isolated from umbilical cords from healthy women in accordance with the ethics guidelines approved by the local Ethics Committee of the University Medical Center Schleswig–Holstein, Kiel, Germany (protocol identification: D518/13) as described further [21]. Cells (seeding density: 20.000—30.000 cells per cm^2^) were cultured in endothelial cell growth medium ECGM (PromoCell, Heidelberg, Germany) supplemented with 4 μL/mL of endothelial cell growth supplement, 0.1 ng/mL epidermal growth factor, 1 ng/mL basic fibroblast growth factor, 90 μg/mL heparin, 1 μg/mL hydrocortisone (all from PromoCell) and 10% fetal bovine serum (Thermo Fisher, Waltham, USA). The cells were maintained in a humidified atmosphere (5% carbon dioxide/95% air) at 37 °C. HUVEC were continuously cultured until passage numbers *P* ≥ 10 (late passage, HUVEC_lp_, *P* ≥ 10). HUVEC with passages *P* < 10 were referred to as early-passage HUVEC (early passage, HUVEC_ep_, *P* < 10). HUVEC_ep_ cultures were utilized at passages four to six, while HUVEC_lp_ cultures were used at passages ten to thirteen.

**Fig. 1:**
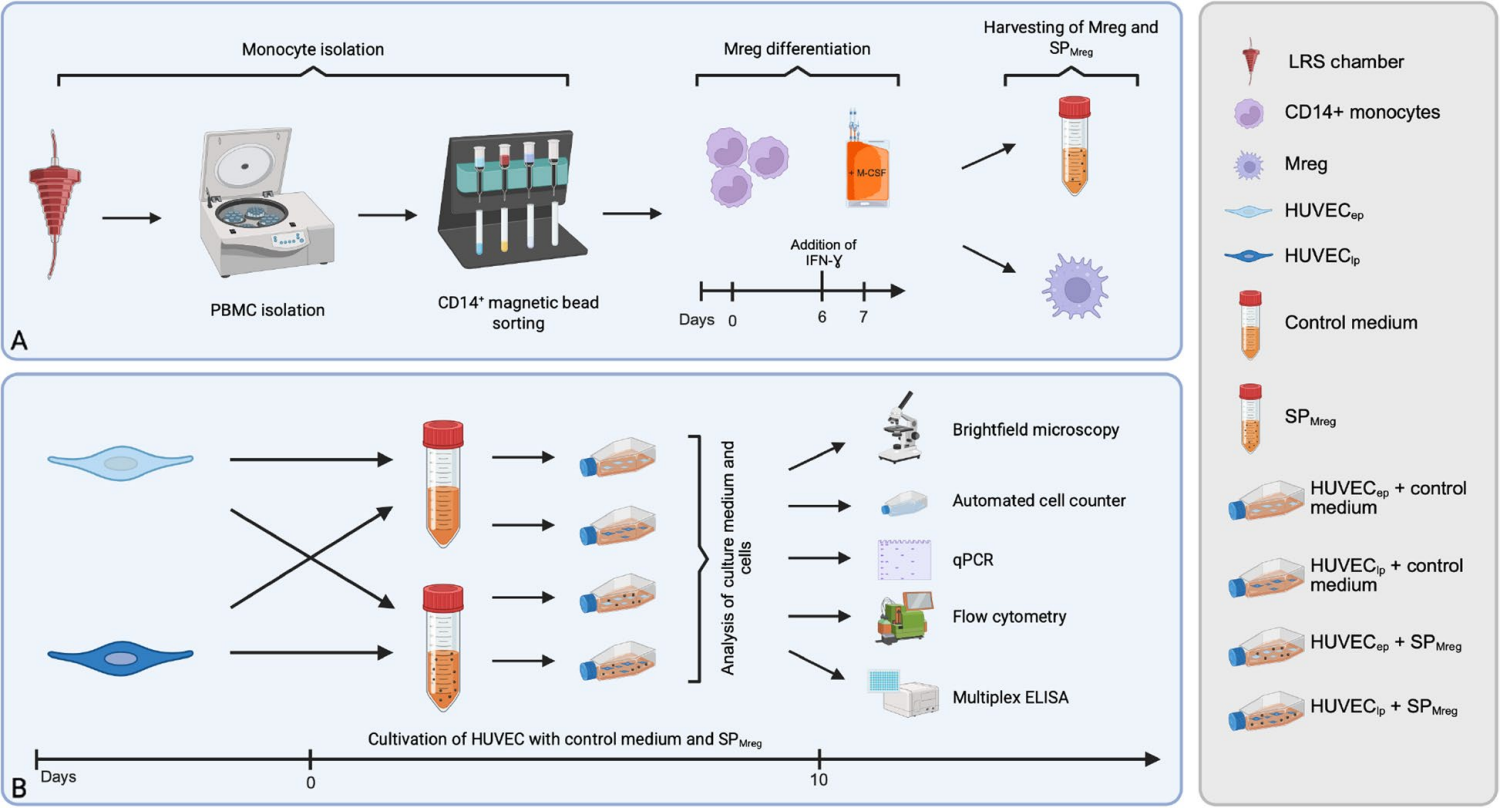
Schematic representation of the manufacturing process of Mreg and experimental setting. **A** Monocytes were obtained from peripheral blood mononuclear cells (PBMC) of healthy donors through density centrifugation and CD14 Magnetic Bead Sorting and cultured for 7 days in cell cultivation bags with the addition of M-CSF for 7 days and IFNγ for 24 h on day 6 to obtain Mreg and SP_Mreg_. **B** HUVEC_ep_ and HUVEC_lp_ from umbilical cords of healthy women were cultured with and without the addition of Mreg supernatants (SP_Mreg_) for 10 days, followed by the analysis of senescence associated factors

HUVEC_ep_ and HUVEC_lp_ were cultured with and without the addition of Mreg-derived secretory products (SP_Mreg_) for 10 days. SP_Mreg_ medium consisted of 50% conditioned Mreg culture medium (RPMI 1640 containing Mreg secretory products) and 50% HUVEC culture mediun (ECGM). The control medium (Co) consisted of 50% non-conditioned Mreg culture medium (RPMI 1640 without Mreg secretory products) and 50% HUVEC culture mediun (ECGM). A detailed overview of the HUVEC passages used and the corresponding SP_Mreg_ batches is provided in the Supplementary Tables 1 and 2. On day 10 cells were harvested by using Accutase for detachment (Innovative Cell Technologies, San Diego, USA) and separated from culture medium by centrifugation (300 × g for 10 min at room temperature). The cells and the conditioned culture media were used for analysis of senescence related parameters (Fig. 1).

### Production of Mreg-derived secretory products (SP_Mreg_)

Monocytes were isolated from eight different leukocyte reduction system (LRS) chambers (labeled Kxxx) provided by the Department of Transfusion Medicine (University Hospital of Schleswig–Holstein, Kiel, Germany) by Ficoll-Paque PLUS gradient centrifugation (GE Healthcare, Chicago, USA) and CD14 positive cell sorting (Miltenyi, Bergisch Gladbach, Germany). Monocyte to Mreg differentiation was achieved by cultivation in differentiation bags (Miltenyi) with RPMI 1640 medium (Gibco, Billings, USA) supplemented with 10% human AB-Serum (Access Biological, Vista, USA) and 4200 IU/ml human M-CSF (R&D Systems, Minneapolis, USA). The culture bags were placed in an incubator under standard culture conditions (humidified atmosphere with 5% carbon dioxide/95% air) at 37 °C. After a 6-day culturing period, 500 IU/ml of human interferon (IFN)γ (R&D Systems, Minneapolis, USA) was added to the cultures and cells were incubated for additional 24 h. On day 7 Mreg were harvested and separated from culture medium (SP_Mreg_) by centrifugation (300 × g for 10 min at room temperature). SP_Mreg_ were stored at – 20 °C until use (Fig. 1).

### Measurement of cell morphology, size and volume

Cell morphology of HUVEC was determined by brightfield microcopy (Leica Microsystems, Wetzlar, Germany). On day 3, 5, 7 and 10 cells were harvested and separated from culture medium by centrifugation (300 × g for 10 min at room temperature) for analysis of cell size and volume by using an automated cell counter (Moxi, Orflo, Ketchum, USA).

### Flow cytometric analysis

Flow cytometric analyses were conducted on day 10 of the cultivation period using the MACS Q10 cytometer from Miltenyi. The specific antibody and the corresponding isotype were directly conjugated with allophycocyanin (APC) for CD105. For evaluation of β-galactosidase positive cells the CellEvent Senescence Green Flow Cytometry Assay Kit was used following the manufacturer’s protocol (Thermo Fisher)*.* For the evaluation of ROS positive cells, a specific cell permeable non-fluorescent probe (Sigma-Aldrich, St. Louis, USA) was applied directly after cell harvesting. For data analyses the MACS-Quantify software for Windows by Miltenyi was used (v2.13.3). The gating strategy consisted of (i) identification of HUVEC based on their size and granularity forward scatter (FSC)/side scatter (SSC) profiles, (ii) exclusion of doublets and non-viable cells (7-AAD nuclear staining, BD Biosciences, #559,925), (iii) identification of HUVEC positive for β-galactosidase, CD105 and ROS.

### Quantitative evaluation of intracellular ROS levels

For the evaluation of the relative intracellular ROS levels a fluorometric assay was performed. HUVEC were seeded in black 96-well plates (5.000 cells/well for HUVEC_ep_ and 10.000 cells/well for HUVEC_lp_) and cultivated with and without the addition of SP_Mreg_ for 10 days. On day 10 a working solution containing 1 mM H_2_DCFDA was added, and excitation (λ = 485 nm) and emission (λ = 535 nm) were measured using the Genios FL Reader (Tecan, Männedorf, Switzerland) every 15 min at 37 °C for 90 min. The increase of fluorescence was calculated using the following formula: $({fluorescence}_{{t}_{x}}-{fluorescence}_{{t}_{0}}/{fluorescence}_{{t}_{0}}) * 100.$  

### PCR-Analysis of CD105

After 10 days of culture HUVEC were harvested and RNA was isolated using the Qiagen RNeasy Mini Kit, following the manufacturer’s protocol (Qiagen, Hilden, Germany) and RNA was transcribed into cDNA as described earlier [16]. The following primers (Metabion, Martinsried, Germany) were synthesized and used to amplify specific fragments of the human transcripts: forward 5’ – GAATTCTGGTACATCTACTCGC – 3’ and reverse 5’—GGCTATGCCATGCTGCTGGTGG – 3’ for CD105, forward 5’ – GTTGGTGGAGCGATTTGTCTGG – 3’ and reverse 5’ – AGGGCAGGGACTTAATCAACGC – 3’ for 18sRNA. All PCR products were separated on 2.5% agarose gels containing 0.005% Roti®-Safe GelStain (Carl Roth, Karlsruhe, Germany) and UV-transillumination was detected using the Vilber Fusion X Spectra reader (Vilber Lourmat, Eberhardzell, Germany). Images of the bands were taken and analyzed with the software ImageJ (v1.41, NIH, Maryland, USA).

### Analysis of SASP associated factors

On day 10 HUVEC were harvested using Accutase and separated from culture medium by centrifugation (300 × g for 10 min at room temperature). Supernatants were aliquoted and stored at −80 °C. Quantification of the SASP factors Activin A, GDF-15, IL-8, PAI-1 and TNF-⍺ were performed by RayBiotec (Georgia, USA) using Multiplex ELISA assays.

### Statistics

The statistics software GraphPad Prism 10.3.1 for windows (GraphPad Software, San Diego, USA) was used for data analyses. Values are expressed as the mean ± standard error of mean (SEM). All data were tested for normality using the Kolmogorov–Smirnov test. In cases normality was not obtained, the data were transformed (CD105 flow cytometry: × 100, log(x); ROS flow cytometry: × 100, log(x), ROS-Assay and SASP Multiplex: 1e, square root of x, arcsin(x)) and analyzed using one-way or two-way ANOVA, or one-sample T-Test. A *P*-value < 0.05 was considered significant.

## Results

### Effects of Mreg-derived secretory products on the morphology of early passage and late passage endothelial cells

**Fig. 2:**
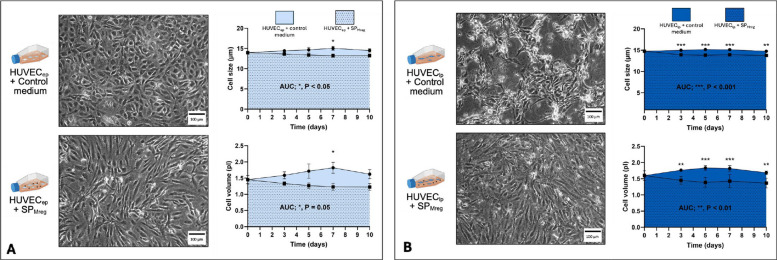
Phenotypic characterization of HUVEC with (+ SP_Mreg_) and without SP_Mreg_ (+ Co). A Phenotypic characterization of HUVEC_ep_ by brightfield microscopy images and determination of the area under the curve for cell size and volume. B Phenotypic characterization of HUVEC_lp_ by brightfield microscopy images and determination of the area under the curve for cells size and volume. Results are presented as mean ± standard error mean (SEM). * *P* < 0.05; ** *P* < 0.01; *** *P* < 0.001. Unpaired T-Test for AUC, 2-way-ANOVA for cell size and volume; HUVEC_ep_ *N* = 7 (P4, P5, P6; HUVEC IX, HUVEC XIII), SP_Mreg_ *N* = 5 (K281, K282, K325, K326, K327); HUVEC_lp_ *N* = 8 (P10, P11, P12, P13; HUVEC IX, HUVEC XIII); SP_Mreg_ *N* = 6 (K277, K279, K281, K282, K289, K326)

HUVEC_ep,_ cultured with control medium, showed a cobblestone like appearance typical for endothelial cells whereas HUVEC_lp_ appeared elongated and more spindle-shaped. HUVEC_lp_ also tended to grow in clusters and did not reach confluence within the defined time period. Under incubation with SP_Mreg_ HUVEC_ep_ as well as HUVEC_lp_ exhibited an elongated and fibroblast-like cell type. Moreover, a comparable confluence of HUVEC_ep_ and HUVEC_lp_ was observed under incubation with SP_Mreg_ (Fig. 2A + B). The area under curve (AUC) for cell size and cell volume was determined for HUVEC_ep_ ± SP_Mreg_ and HUVEC_lp_ ± SP_Mreg_ and showed a significant reduction of cell size and volume especially in HUVEC_lp_ + SP_Mreg_ (AUC cell size: HUVEC_ep_ + Co: 133.8 ± 4.309 µm x days; *P* < 0.05 vs. HUVEC_ep_ + SP_Mreg_: 122.8 ± 3.289 µm x days; HUVEC_lp_ + Co: 149.9 ± 1.786 µm x days; *P* < 0.001 vs. HUVEC_lp_ + SP_Mreg_: 140.0 ± 1.985 µm x days; AUC cell volume: HUVEC_ep_ + Co: 16.56 ± 1.474 pl x days; P = 0.05 vs. HUVEC_ep_ + SP_Mreg_: 12.97 ± 0.944 pl x days; HUVEC_lp_ + Co: 17.55 ± 0.6421 pl x days; *P* < 0.01 vs. HUVEC_lp_ + SP_Mreg_: 14.40 ± 0.572 pl x days; Fig. 2 A + B).

### Effects of Mreg-derived secretory products on CD105 expression by early passage and late passage endothelial cells

**Fig. 3:**
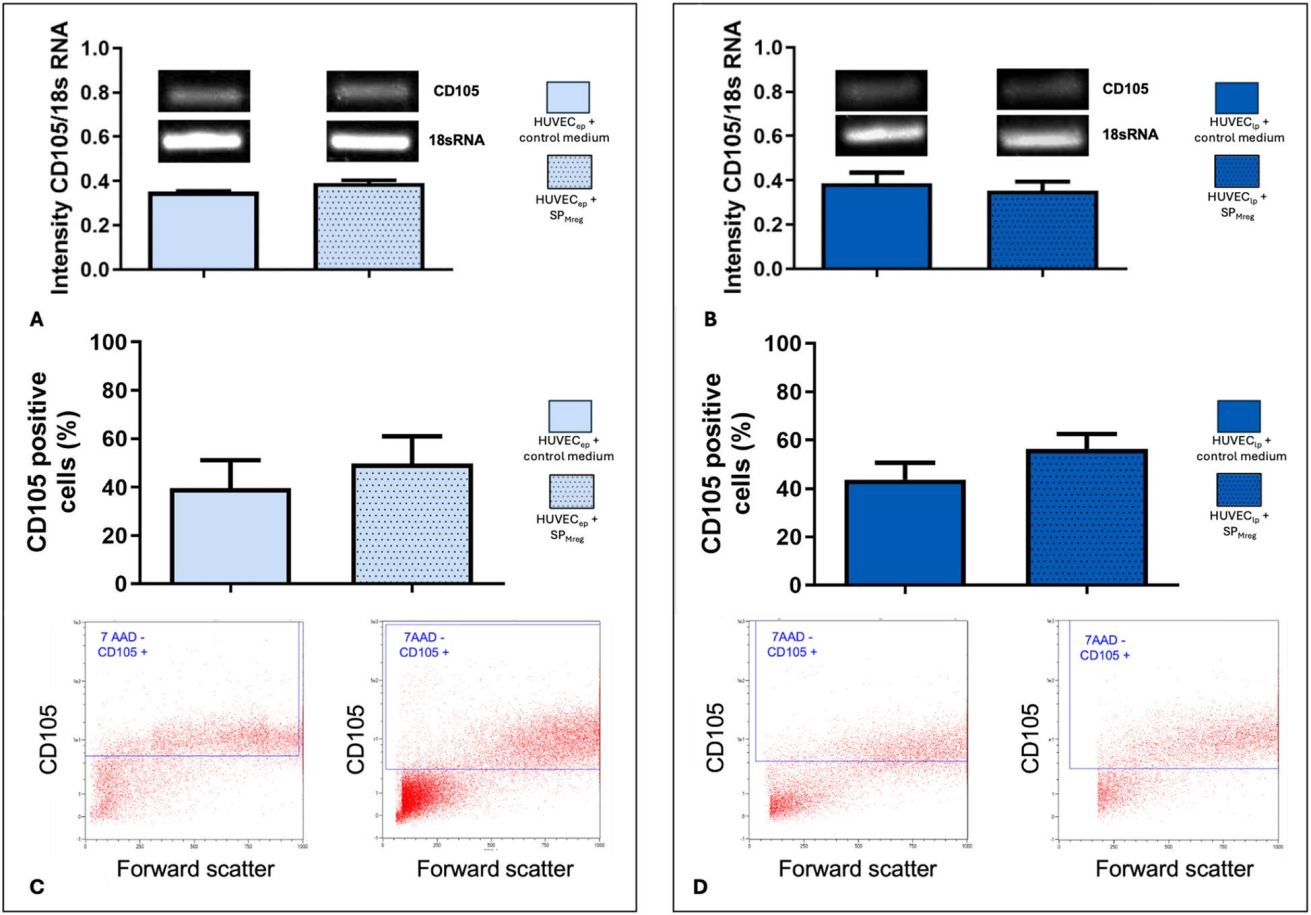
Characterization of CD105 expression in HUVEC_ep_ and HUVEC_lp_ after the 10-day cultivation period with (+ SP_Mreg_) and without SP_Mreg_ (+ Co). A + B qPCR analysis of HUVEC_ep_ and HUVEC_lp_. C + D Flow cytometric analysis of HUVEC_ep_ and HUVEC_lp_. Results are presented as mean ± standard error mean (SEM). One-sample T-Test, transformation of CD105 flow cytometric analysis: log(x); HUVEC_ep_ *N* = 7 for flow cytometric analysis (P4, P5, P6; HUVEC IX, HUVEC XIII), SP_Mreg_ *N* = 5 (K281, K282, K325, K326, K327) and *N* = 3 for PCR of CD105 (P4, P5; HUVEC XIII), SP_Mreg_ *N* = 3 (K325, K326, K327); HUVEC_lp_ *N* = 8 for flow cytometric analysis (P10, P11, P12, P13; HUVEC IX, HUVEC XIII); SP_Mreg_ *N* = 6 (K277, K279, K281, K282, K289, K326) and *N* = 3 for PCR of CD105 (P10; HUVEC XIII), SP_Mreg_ *N* = 2 (K289, K326)

To evaluate the effect of SP_Mreg_ on CD105 gene expression semiquantitative analysis was performed. CD105 expression did not differ in qPCR analysis between cultures of HUVEC_ep_ nor HUVEC_lp_ with and without the addition of SP_Mreg_ (HUVEC_ep_ + Co: 0.350 ± 0.002; *P* > 0.05 vs. HUVEC_ep_ + SP_Mreg_ 0.390 ± 0.013; HUVEC_lp_ + Co: 0.386 ± 0.047; *P* > 0.05 vs. HUVEC_lp_ + SP_Mreg_: 0.353 ± 0.040; Fig. 3A + B). For evaluation of the number of CD105 positive cells flow cytometric analysis was performed. Although the number of CD105 positive HUVEC_ep_ and HUVEC_lp_ tended to increase upon addition of SP_Mreg_, the differences did not reach statistical significance (HUVEC_ep_ + Co: 39.55 ± 11.49%; *P* > 0.05 vs. HUVEC_ep_ + SP_Mreg_: 49.62 ± 11.35%; HUVEC_lp_ + Co: 43.76 ± 6.838%; *P* > 0.05 vs. HUVEC_lp_ + SP_Mreg_: 56.32 ± 6.153%; Fig. 3A - D).

### Effects of Mreg-derived secretory products on β-galactosidase and reactive oxygen species (ROS) in early passage and late passage endothelial cells

**Fig. 4:**
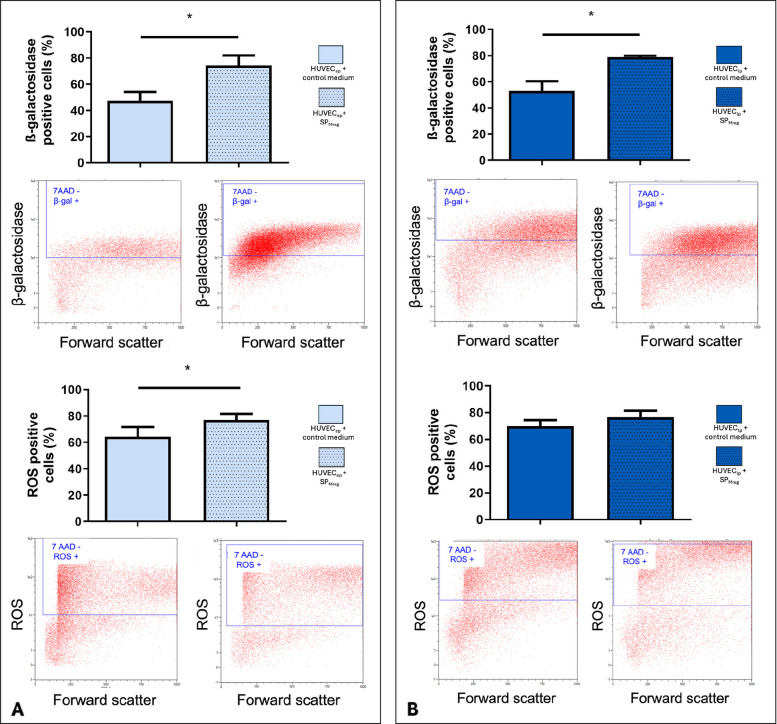
Analysis of the percentage of β-galactosidase and ROS positive HUVEC_ep_ and HUVEC_lp_ after the 10-day cultivation period with (+ SP_Mreg_) and without SP_Mreg_ (+ Co). A Flow cytometric analysis of β-galactosidase and ROS positive HUVEC_ep_. B Flow cytometric analysis of β-galactosidase and ROS positive HUVEC_lp_. Results are presented as mean ± standard error mean (SEM). * *P* < 0.05. One-sample T-Test, transformation of ROS flow cytometric analysis: log(x); HUVEC_ep_ *N* = 7 (P4, P5, P6; HUVEC IX, HUVEC XIII), SP_Mreg_ *N* = 5 (K281, K282, K325, K326, K327); HUVEC_lp_ *N* = 8 (P10, P11, P12, P13; HUVEC IX, HUVEC XIII); SP_Mreg_ *N* = 6 (K277, K279, K281, K282, K289, K326)

Flow cytometric analysis of senescence related factors showed a significant increase of β-galactosidase and ROS positive HUVEC_ep_ under SP_Mreg_ treatment (β-Galactosidase: HUVEC_ep_ + Co: 47.30 ± 6.670%; *P* < 0.05 vs. HUVEC_ep_ + SP_Mreg_: 74.21 ± 7.225%; ROS: HUVEC_ep_ + Co: 64.20 ± 7.399%; *P* < 0.05 vs. HUVEC_ep_ + SP_Mreg_: 77.11 ± 4.512%; Fig. 4A). SP_Mreg_ also caused a significant increase in the number of β-galactosidase positive cells in HUVEC_lp_ (HUVEC_lp_ + Co: 52.97 ± 7.421%; *P* < 0.05 vs. HUVEC_lp_ + SP_Mreg_: 78.85 ± 1.163%; Fig. 4B), but did not alter the number of ROS positive cells (HUVEC_lp_ + Co: 69.77 ± 4.536%; *P* > 0.05 vs. HUVEC_lp_ + SP_Mreg_ 76.39 ± 5.001%; Fig. 4B).

**Fig. 5:**
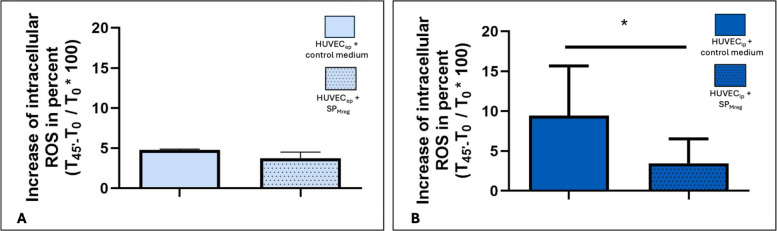
Characterization of relative intracellular changes of ROS levels in HUVEC_ep_ and HUVEC_lp_ after the 10-day cultivation period with (+ SP_Mreg_) and without SP_Mreg_ (+ Co). A HUVEC_ep_. B HUVEC_lp_. Results are presented as mean ± standard error mean (SEM). * *P* < 0.05. One-sample T-Test, transformation: square root of x, arcsin(x); HUVEC_ep_ *N* = 3 (P4, P5; HUVEC XIII), SP_Mreg_ *N* = 3 (K325, K326, K327); HUVEC_lp_ *N* = 3 (P10; HUVEC XIII), SP_Mreg_ *N* = 2 (K289, K326)

HUVEC were incubated with H_2_DCFDA to evaluate the oxidative status of the cells. In HUVEC_ep_ addition of SP_Mreg_ did not change the increase of intracellular ROS levels over the time period of 45 min (HUVEC_ep_ + Co: 4.773 ± 0.111; *P* > 0.05 vs. HUVEC_ep_ + SP_Mreg_: 3.753 ± 0.751; Fig. 5A). However, applying SP_Mreg_ significantly attenuated the intracellular increase of ROS in HUVEC_lp_ (HUVEC_lp_ + Co: 9.42 ± 6.241; *P* < 0.05 vs. HUVEC_lp_ + SP_Mreg_: 3.45 ± 3.061; Fig. 5B).

### Effects of Mreg-derived secretory products on SASP factor secretion in cultures of early passage and late passage endothelial cells

**Fig. 6:**
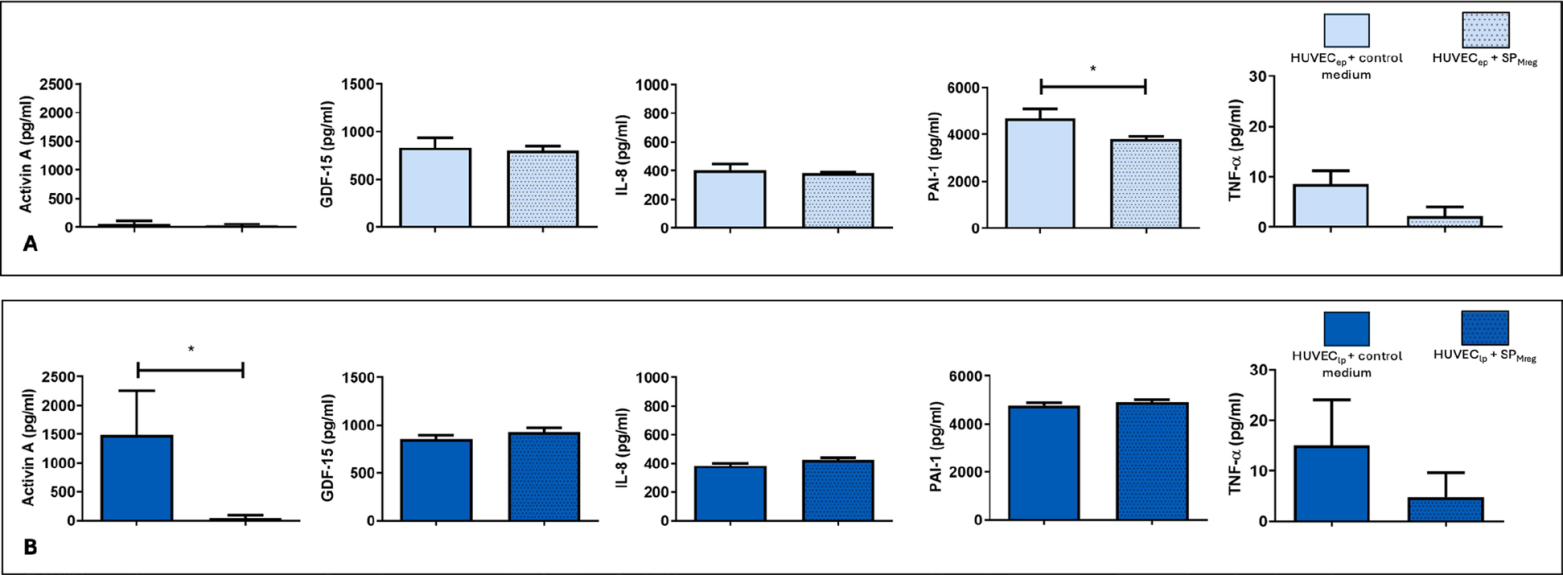
Senescence associated secretory phenotype (SASP) factor protein secretion in HUVEC_ep_ and HUVEC_lp_ after the 10-day cultivation period with (+ SP_Mreg_) and without SP_Mreg_ (+ Co). **A** HUVEC_ep_. **B** HUVEC_lp_. Results are presented as mean ± standard error mean (SEM). * *P* < 0.05; One-sample T-Test, transformation: square root of x, arcsin(x); HUVEC_ep_*N* = 3 (P5, P6; HUVEC IX), SP_Mreg_*N* = 2 (K281, K282); HUVEC_lp_*N* = 3 (P11, P12, P13; HUVEC IX), SP_Mreg_*N* = 2(K289, K326)

To analyze the effects of SP_Mreg_ on SASP (senescence associated secretory phenotype) factor secretion of HUVEC_ep_ and HUVEC_lp,_ multiplex ELISAs were performed. In HUVEC_ep_ cultures, SP_Mreg_ resulted in significantly lower PAI-1 protein secretion (HUVEC_ep_ + Co: 4686 ± 413.8 pg/ml; *P* < 0.05 vs. HUVEC_ep_ + SP_Mreg_: 3808 ± 103.3 pg/ml; Fig. 6A), but did not have a significant effect on the secretion of the SASP factors Activin A, GDF-15, IL-8 and TNF-⍺ (Supplementary Table 3). In contrast, cultivation with SP_Mreg_ led to a significant decrease of Activin A protein secretion in HUVEC_lp_ (HUVEC_lp_ + Co: 1487.10 ± 628.97 pg/ml; *P* < 0.05 vs. HUVEC_lp_ + SP_Mreg_: 47.93 ± 39.14 pg/ml; Fig. 6B) while no significant differences in protein secretion of GDF-15, IL-8, PAI-1 and TNF-⍺ were observed (Supplementary Table 3).

Statistical comparisons between HUVEC_ep_ and HUVEC_lp_ with respect to all analyzed parameters under basal conditions, as well as with and without SP_Mreg_ are presented in Supplementary Figs. 1–4.

## Discussion

Cardiovascular diseases (CVDs) are the leading cause of mortality worldwide and within the demographic change a constant search for new preventive and therapeutic strategies started in the last decades [1]. Cardiovascular aging and cellular senescence, especially of endothelial cells, plays a pivotal role in the onset and progression of CVDs by the induction of a chronic, sterile inflammation [6]. Macrophages are essential regulators of tissue homeostasis, immune defense and inflammation but their effects on surrounding tissues strongly depend on their *in-vivo* polarization [22, 23]. To simplify, inflammatory M1 and anti-inflammatory M2 macrophages can be distinguished, but the *in-vivo* phenotypes are more complex and dynamically regulated by the surrounding micro-environment [24]. In recent years different research groups have shown that *in-vitro* generated regulatory macrophages (Mreg) possess anti-inflammatory and immunosuppressive effects on surrounding tissues [17, 25]. Mreg are considered to present a M2-subtype of macrophages that might also be able to positively influence endothelial cell senescence by limiting the induction of a chronic, sterile inflammation [19, 26].

The present study investigates the effects of secretory products derived from IFN-γ (day 6) induced Mreg (SP_Mreg_) on cellular senescence markers in early-passage (HUVEC_ep_) and late-passage (HUVEC_lp_) of human endothelial cells, serving as a model for the aging endothelium associated with the development of cardiovascular diseases (CVDs). In previous work, we showed that Mreg generated using a slightly different protocol with an early IFN-γ stimulation at day 0 (Mreg_IFNγ0_) differ substantially from classical late IFN-γ induced Mreg, exhibiting a more M1-like and pro-inflammatory phenotype as well as marked alterations in their secretome [27]. Given that classical late IFN-γ induced Mreg are predominantly anti-inflammatory, whereas Mreg_IFNγ0_ display a more inflammatory profile—and that variations in IFN-γ timing or omission are likely to generate additional Mreg subtypes – we chose to focus primarily on the effects of SP_Mreg_ from well-characterized classical IFN-γ-induced Mreg in the present study.

For induction of cellular aging, HUVEC were passaged until a critical passage number (*P* ≥ 10), resulting in cessation of proliferative capacity [28]. The resulting HUVEC_lp_ exhibited morphological characteristics typical for aged endothelial cells, including a heterogeneous appearance and an elongated, flattened cell body [29].

When cultured with SP_Mreg_, both HUVEC_ep_ and HUVEC_lp_ adopted an elongated, fibroblast-like morphology, in contrast to the typical cobblestone appearance of HUVEC_ep_ in control medium. Additionally, SP_Mreg_ treatment led to a significant reduction in cell size and volume especially in HUVEC_lp_. Changes in endothelial cell morphology are not only linked to cellular aging but also affect their secretory profile [30]. Spindle-shaped endothelial cells are considered anti-inflammatory in the development of atherosclerotic lesions by inhibiting monocyte secretion [31]. Furthermore, increased cell size and volume are hallmark features of cellular senescence and linked to shifts in protein concentration as well as metabolic activity that drive the onset of the senescent phenotype [32]. Our findings that SP_Mreg_ treatment led to a significant reduction in cell size and volume, particularly in late passage HUVEC_lp_ highlight the potential of SP_Mreg_ to counteract senescence-associated morphological changes and suggest that the premature induction and progression of senescence, especially in aging endothelial cells, may be influenced by the Mreg secretory factors.

Regarding the endothelial senescence marker CD105, no statistically significant differences were observed in either HUVEC_ep_ or HUVEC_lp_ following SP_Mreg_ treatment, although there was a slight trend towards increased CD105 positivity in both cell types. CD105 is a component of the TGF-β receptor complex and plays a key role in regulating cell growth, migration, angiogenesis, vascular tone and extracellular matrix production [33, 34]. Elevated CD105 levels are associated not only with cellular senescence but also with endothelial cell activation, immune cell adhesion to the endothelium, chronic inflammation and ultimately the development and progression of atherosclerotic lesions [34, 35]. By maintaining CD105 at a steady level the addition of Mreg secretory factors may exert a beneficial effect on the endothelium, potentially limiting inflammation and plaque formation.

SP_Mreg_ treatment resulted in a significant increase in β-galactosidase activity in both HUVEC_ep_ and HUVEC_lp_. β-galactosidase is considered as a marker of cellular senescence *in-vitro* and is therefore referred to as senescence associated β-galactosidase (SA-β-galactosidase) [36]. SA-β-galactosidase is produced in lysosomes and its expression is correlated with lysosomal abundance [37]. However, recent studies have demonstrated that the *in-vitro* expression of β-galactosidase can also be induced by alternative conditions, such as contact inhibition or the absence of growth factors, and that β-galactosidase is not strictly required for the induction or maintenance of senescence [37, 38]. In summary, the finding that β-galactosidase is upregulated in HUVEC_ep_ and HUVEC_lp_ following SP_Mreg_ treatment is somewhat unexpected. It remains unclear whether this reflects a specific senescence-associated effect or if other culture conditions, as previously discussed, contribute to this observation.

Treatment with SP_Mreg_ also led to a significant increase in the percentage of ROS-positive cells in HUVEC_ep_ cultures. In contrast, the increase of intracellular ROS levels over time in HUVEC_lp_ was significantly lower under SP_Mreg_ treatment. Reactive oxygen species are highly reactive molecules physiologically generated during aerobic metabolism. Their production is tightly regulated to prevent oxidative stress and cellular damage [39]. In senescent cells mitochondrial dysfunction can lead to excessive ROS accumulation triggering senescence associated secretory phenotype (SASP) factor production and promoting senescence in neighboring cells [40]. Moreover, elevated ROS levels are linked to endothelial dysfunction and promote the development of atherosclerosis, hypertension and severe cardiovascular events by inhibiting nitric oxide (NO) production, inducing vasoconstriction and activating the coagulation cascade [41, 42]. The finding that HUVEC_lp_ cultured with Mreg secretory products exhibit a significantly lower increase in intracellular ROS levels over time compared to those cultured with control medium supports the hypothesis that Mreg derived supernatants may exert protective effects on the aging endothelium.

To investigate whether Mreg secretory products also modulate SASP factor realease in endothelial cells, conditioned culture media were characterized using Multiplex assays. In HUVEC_ep_ SP_Mreg_ treatment significantly reduced the secretion of Plasminogen activator inhibitor 1 (PAI-1) but had no significant impact on other SASP factors. In HUVEC_lp,_ treatment with SP_Mreg_ significantly decreased Activin A release, whereas no significant changes were observed on other SASP components. Elevated PAI-1 levels are associated with pro-fibrotic and pro-thrombotic phenotypes by reducing fibrinolytic activity, a phenomenon commonly seen in aging individuals [43, 44]. By lowering PAI-1 levels in young endothelial cells Mreg secretory products may act as a protector of the vascular endothelium, potentially reducing age-related vascular dysfunction. Activin A, a member of the TGF-β superfamily, regulates cell growth, differentiation, apoptosis and angiogenesis in endothelial cells and other cell types [45]. Elevated levels of Activin A have been linked to endothelial senescence *in-vitro* and endothelial dysfunction, aging and age-associated CVDs *in-vivo*. The detrimental effects of Activin A are primarily mediated through the induction of oxidative stress and upregulation of adhesion molecule expression in endothelial cells [46, 47]. By suppressing Activin A production Mreg secretory products could exert vasoprotective effects on the aging endothelium.

**Fig. 7:**
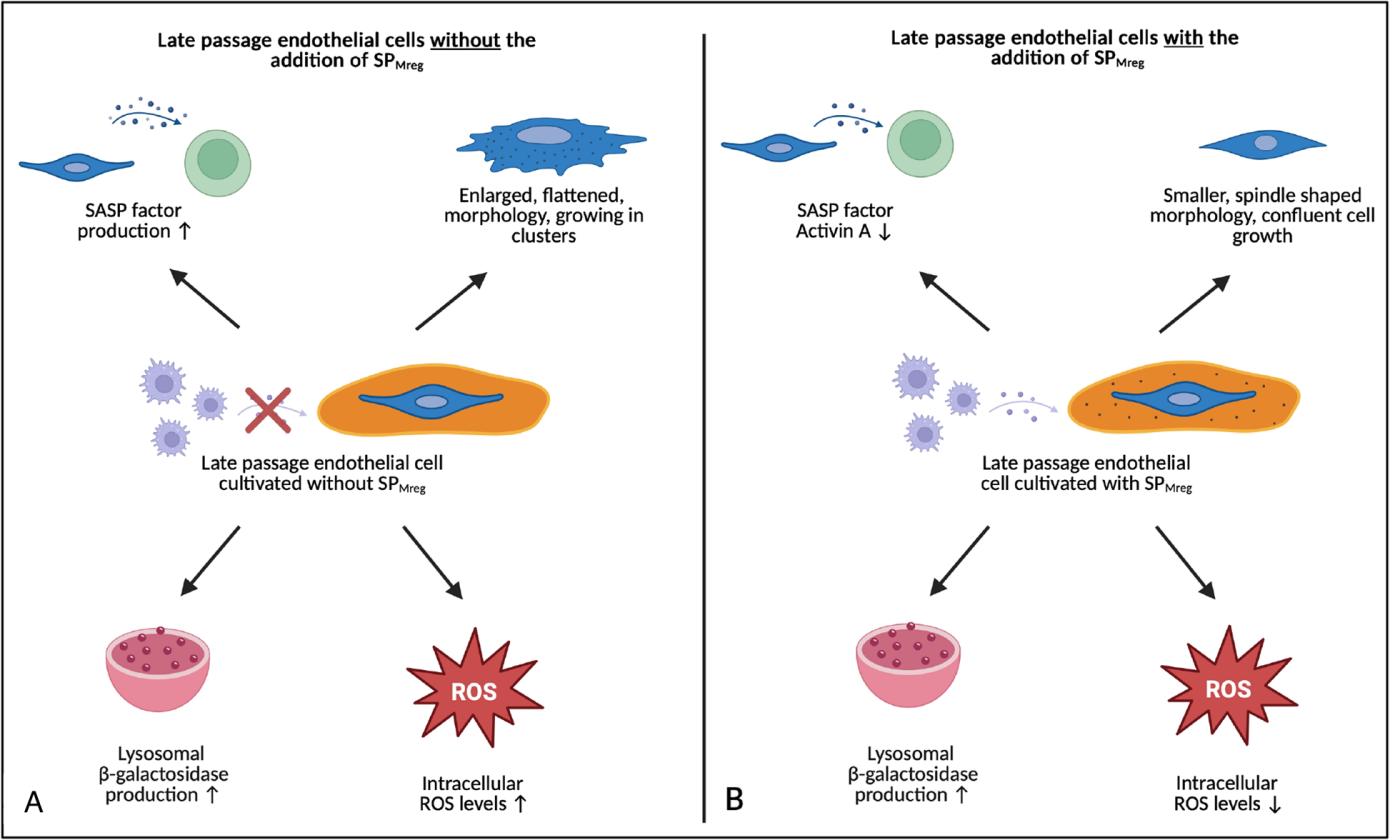
Key outcomes of this study at a glance. Comparison of late passage HUVEC (HUVEC_lp_) cultured without (+ Co) (**A**) and with (**B**) the addition of SP_Mreg_

While this study offers important insights into the effects of regulatory macrophage secretory products on cardiovascular aging and endothelial cell aging (Fig. 7), several limitations should be acknowledged. First, HUVEC with replicative senescence serve as a practical *in-vitro* model but may not fully reflect the complexity of vascular aging in living organisms. Future studies including adult arterial endothelial cells to validate the generalizability of our findings or *in-vivo* approaches would enhance translational relevance. Second, the passage number difference between HUVEC_ep_ and HUVEC_lp_ was relatively narrow (4–9 passages), which may explain why some senescence-associated parameters did not differ significantly despite established indicators of replicative senescence such as increased Activin A secretion and altered cell morphology. Third, in this study, senescence was characterized using multiple markers, including cell morphology and size, β-galactosidase activity, CD105 expression, reactive oxygen species (ROS), and specific components of the senescence-associated secretory phenotype (SASP). Given the complexity of cellular senescence, the inclusion of additional markers such as p16^INK4a^ or p21^CIP1/WAF1^ in future studies could further strengthen the characterization and provide a more comprehensive understanding of the senescent phenotype of endothelial cells. Forth, while the study demonstrates significant senescence relevant effects of Mreg secretory products on endothelial cells, the specific bioactive components responsible for these effects have yet to be identified. Further characterization of the Mreg secretome, along with mechanistic and in vivo experiments, will be essential to deepen our understanding of the underlying pathways and to optimize potential therapeutic applications. As an initial basis for future studies aimed at identifying the responsible factors, previous work from our group characterizing the Mreg secretome may provide valuable preliminary insights [27, 48, 49].

In summary, our findings suggest that Mreg secretory products can modulate endothelial cell morphology as well as key markers associated with cellular senescence and cardiovascular aging, which may have important implications for vascular health during aging. These results underscore the potential of Mreg and their secretome as potential candidates for preventive or therapeutic strategies in cardiovascular aging and cardiovascular diseases. Nonetheless, further research is needed to elucidate the underlying mechanisms, identify the active components within the Mreg secretome, and compare their effects with other macrophage subtypes.

## Supplementary Information

Supplementary Material 1.

Supplementary Material 2.

Supplementary Material 3.

## Data Availability

The datasets analyzed during the current study are available from the corresponding author on reasonable request.
