# Supplementary material for: Secretory products from regulatory macrophages modulate senescence in human endothelial cells: implications for cardiovascular aging and diseases
Source: BMC Cardiovasc Disord. 2026 Mar 31;26:301. doi: 10.1186/s12872-026-05732-w (PMC13063746; doi:10.1186/s12872-026-05732-w)
Supplement: Supplementary file 1 — Supplementary Material 1. [file 12872_2026_5732_MOESM1_ESM.pptx]

## Slide 1
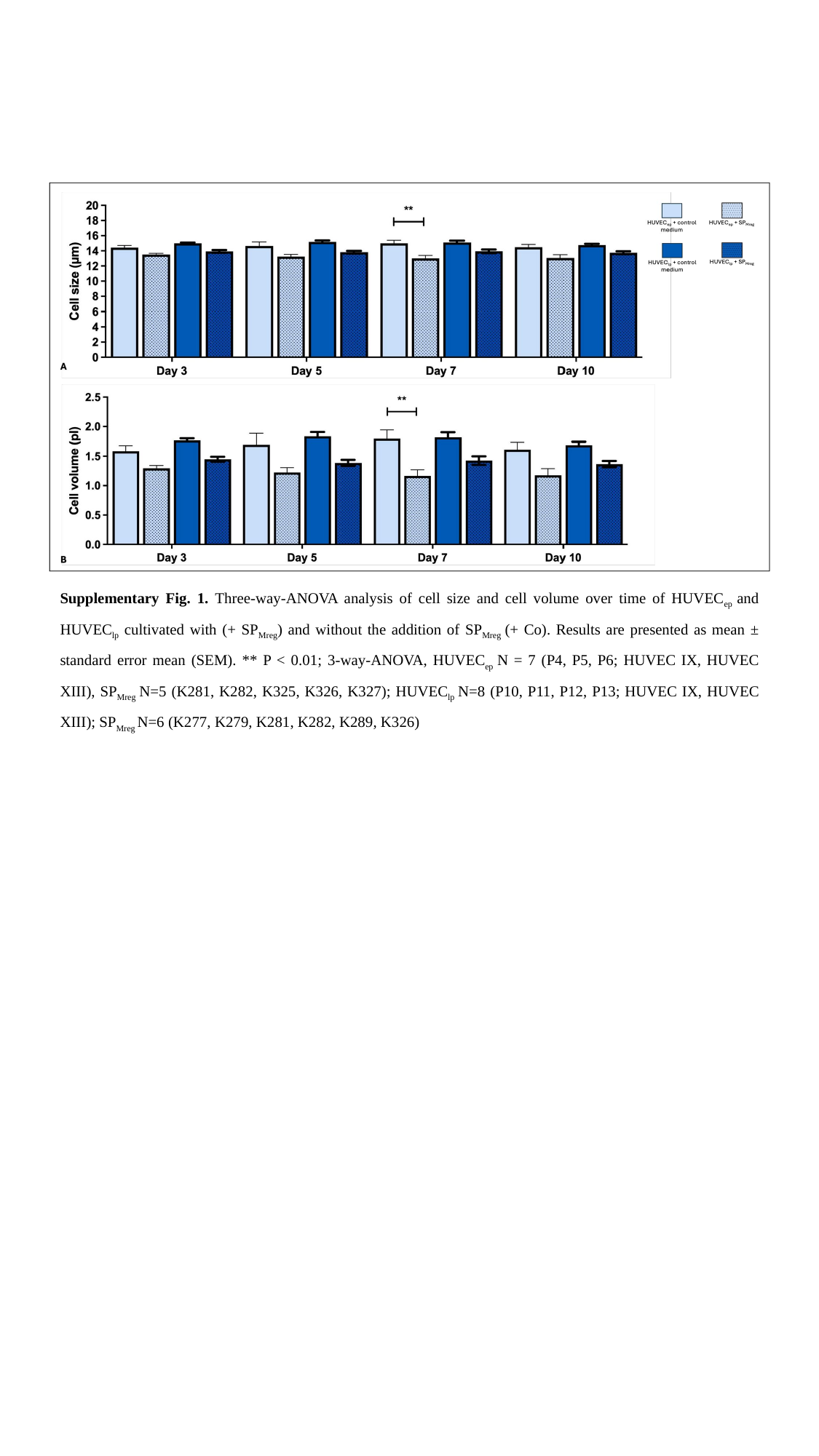

Supplementary Fig. 1. Three-way-ANOVA analysis of cell size and cell volume over time of HUVECep and HUVEClp cultivated with (+ SPMreg) and without the addition of SPMreg (+ Co). Results are presented as mean ± standard error mean (SEM). ** P < 0.01; 3-way-ANOVA, HUVECep N = 7 (P4, P5, P6; HUVEC IX, HUVEC XIII), SPMreg N=5 (K281, K282, K325, K326, K327); HUVEClp N=8 (P10, P11, P12, P13; HUVEC IX, HUVEC XIII); SPMreg N=6 (K277, K279, K281, K282, K289, K326)

## Slide 2
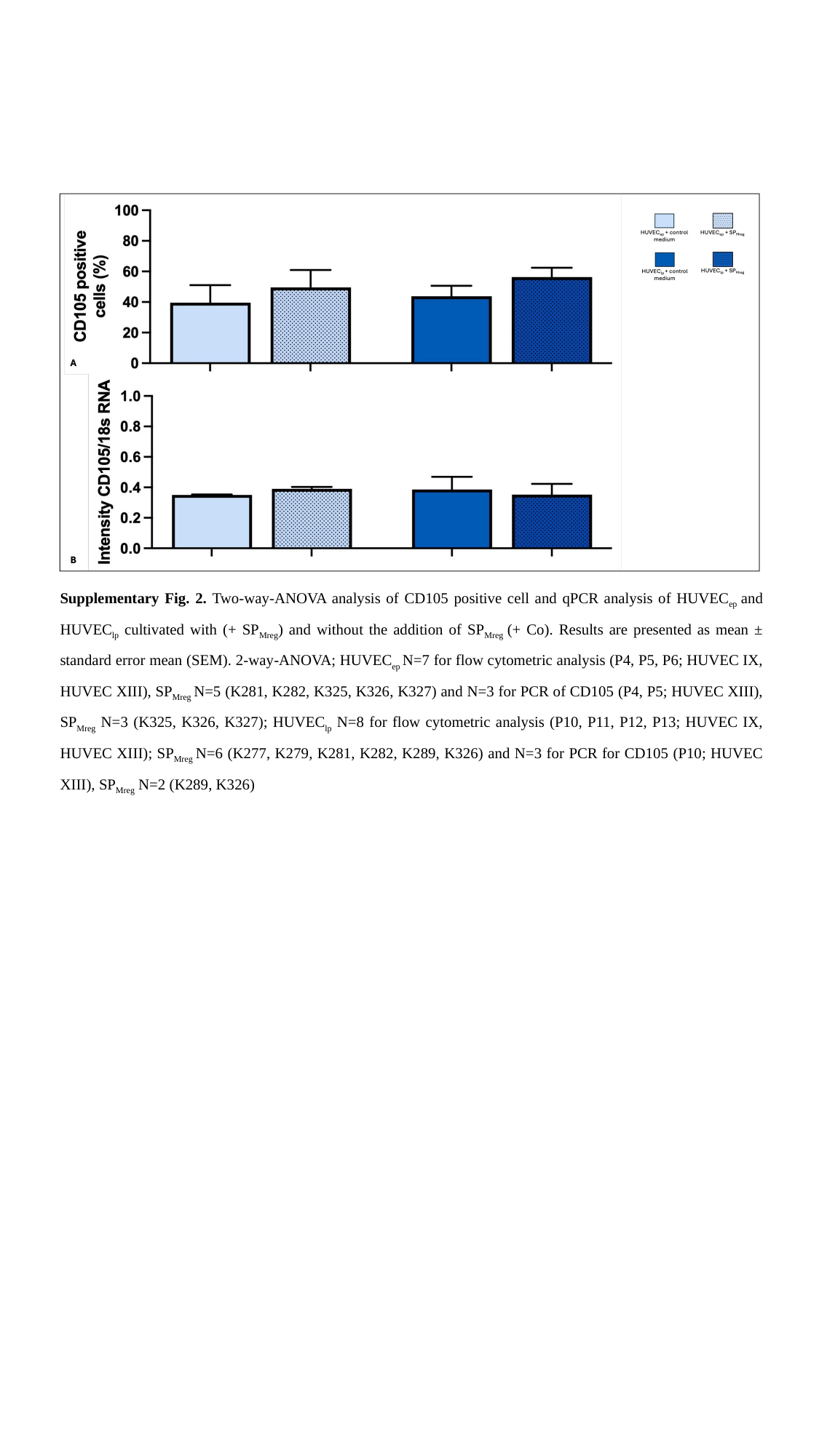

Supplementary Fig. 2. Two-way-ANOVA analysis of CD105 positive cell and qPCR analysis of HUVECep and HUVEClp cultivated with (+ SPMreg) and without the addition of SPMreg (+ Co). Results are presented as mean ± standard error mean (SEM). 2-way-ANOVA; HUVECep N=7 for flow cytometric analysis (P4, P5, P6; HUVEC IX, HUVEC XIII), SPMreg N=5 (K281, K282, K325, K326, K327) and N=3 for PCR of CD105 (P4, P5; HUVEC XIII), SPMreg N=3 (K325, K326, K327); HUVEClp N=8 for flow cytometric analysis (P10, P11, P12, P13; HUVEC IX, HUVEC XIII); SPMreg N=6 (K277, K279, K281, K282, K289, K326) and N=3 for PCR for CD105 (P10; HUVEC XIII), SPMreg N=2 (K289, K326)

## Slide 3
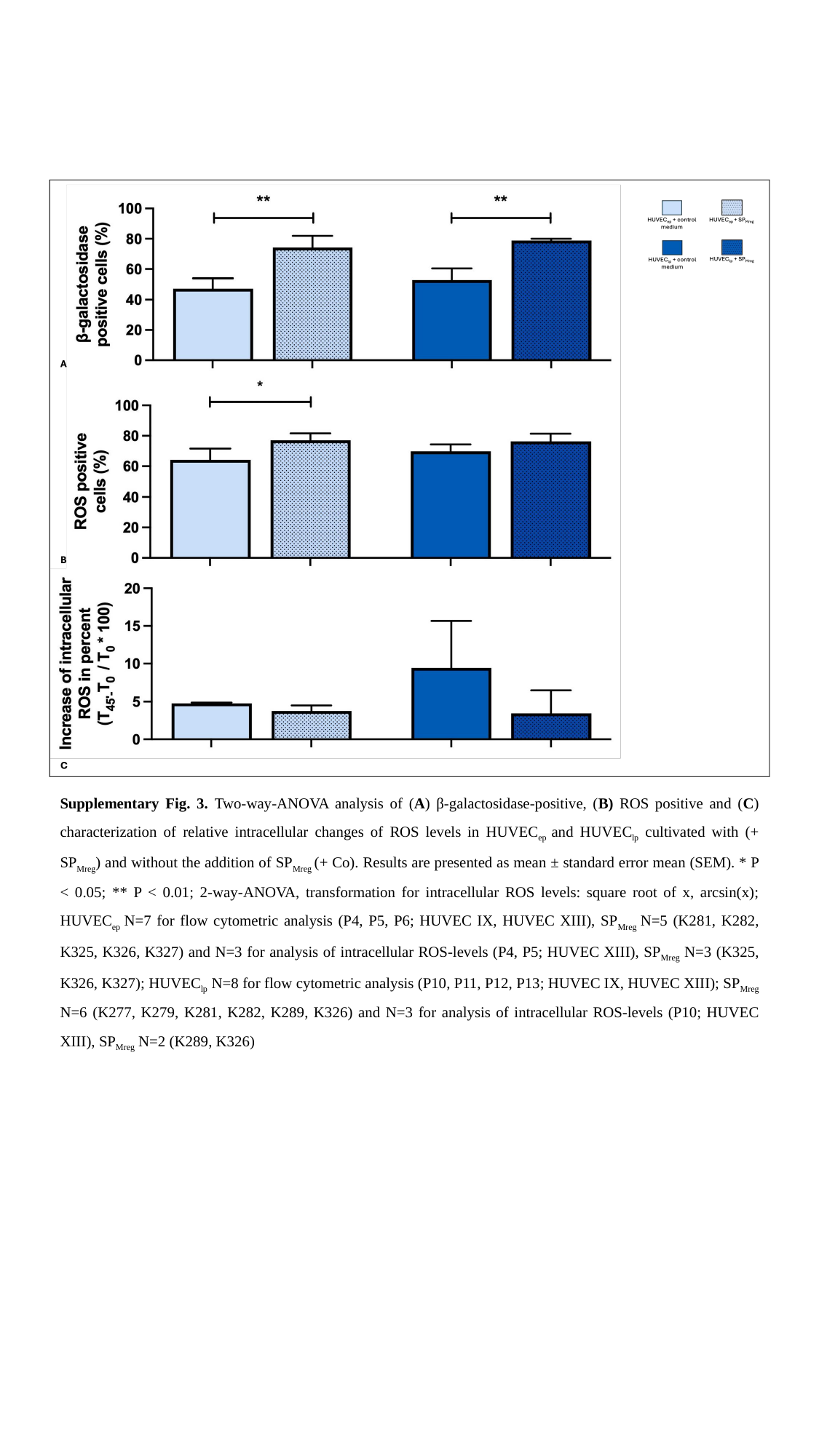

Supplementary Fig. 3. Two-way-ANOVA analysis of (A) β-galactosidase-positive, (B) ROS positive and (C) characterization of relative intracellular changes of ROS levels in HUVECep and HUVEClp cultivated with (+ SPMreg) and without the addition of SPMreg (+ Co). Results are presented as mean ± standard error mean (SEM). * P < 0.05; ** P < 0.01; 2-way-ANOVA, transformation for intracellular ROS levels: square root of x, arcsin(x); HUVECep N=7 for flow cytometric analysis (P4, P5, P6; HUVEC IX, HUVEC XIII), SPMreg N=5 (K281, K282, K325, K326, K327) and N=3 for analysis of intracellular ROS-levels (P4, P5; HUVEC XIII), SPMreg N=3 (K325, K326, K327); HUVEClp N=8 for flow cytometric analysis (P10, P11, P12, P13; HUVEC IX, HUVEC XIII); SPMreg N=6 (K277, K279, K281, K282, K289, K326) and N=3 for analysis of intracellular ROS-levels (P10; HUVEC XIII), SPMreg N=2 (K289, K326)

## Slide 4
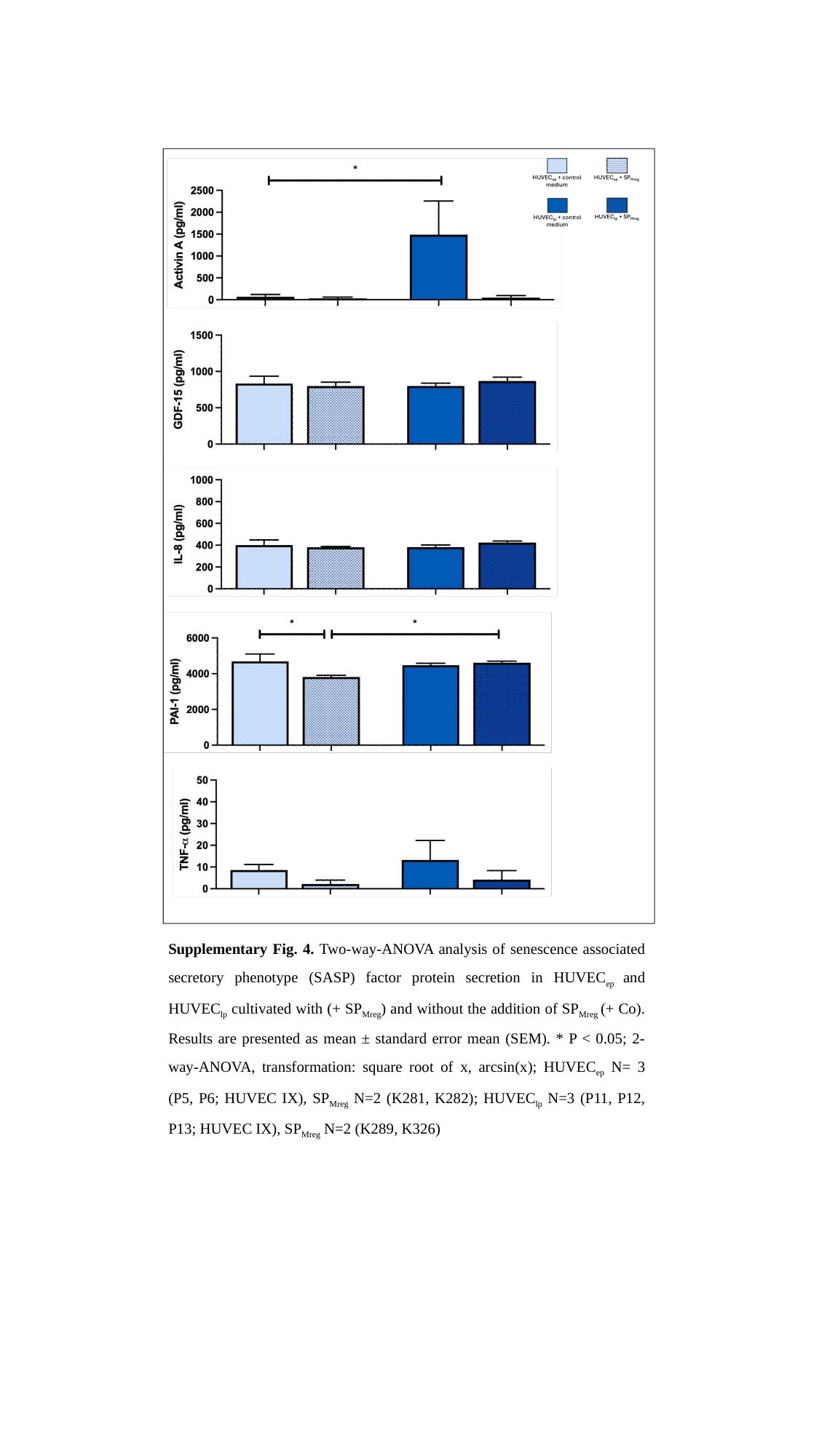

Supplementary Fig. 4. Two-way-ANOVA analysis of senescence associated secretory phenotype (SASP) factor protein secretion in HUVECep and HUVEClp cultivated with (+ SPMreg) and without the addition of SPMreg (+ Co). Results are presented as mean ± standard error mean (SEM). * P < 0.05; 2-way-ANOVA, transformation: square root of x, arcsin(x); HUVECep N= 3 (P5, P6; HUVEC IX), SPMreg N=2 (K281, K282); HUVEClp N=3 (P11, P12, P13; HUVEC IX), SPMreg N=2 (K289, K326)
