# Supplementary material for: Secretory products from regulatory macrophages modulate senescence in human endothelial cells: implications for cardiovascular aging and diseases
Source: BMC Cardiovasc Disord. 2026 Mar 31;26:301. doi: 10.1186/s12872-026-05732-w (PMC13063746; doi:10.1186/s12872-026-05732-w)
Supplement: Supplementary file 2 — Supplementary Material 2. [file 12872_2026_5732_MOESM2_ESM.docx]

| Activin A: HUVEC_ep_ + Co: 63.95 ± 53.6 pg/ml; P > 0.05 vs. HUVEC_ep_ + SP_Mreg_: 28.37 ± 29.37 pg/ml |
| --- |
| GDF-15: HUVEC_ep_ + Co: 834.5 ± 100.3 pg/ml; P > 0.05 vs HUVEC_ep_ + SP_Mreg_: 798.9 ± 53.49 pg/ml |
| IL-8: HUVEC_ep_ + Co: 400.9 ± 47.18 pg/ml; P > 0.05 vs. HUVEC_ep_ + SP_Mreg_: 380.9 ± 7.289 pg/ml |
| TNF-⍺: HUVEC_ep_ + Co: 8.590 ± 2.542 pg/ml; P > 0.05 vs. HUVEC_ep_ + SP_Mreg_: 2.207 ± 1.753 pg/ml |
| GDF-15: HUVEC_lp_ + Co: 799.9 ± 38.65 pg/ml; P > 0.05 vs. HUVEC_lp_ + SP_Mreg_: 868.5 ± 52.55 pg/ml |
| IL-8: HUVEC_lp_ + Co: 382.8 ± 32.84 pg/ml; P > 0.05 vs. HUVEC_lp_ + SP_Mreg_: 424.5 ± 25.16 pg/ml |
| PAI-1: HUVEC_lp_ + Co: 4474 ± 109.9 pg/ml; P > 0.05 vs. HUVEC_lp_ + SP_Mreg_: 4621 ± 89.1 pg/ml |
| TNF-⍺: HUVEC_lp_ + Co: 13.24 ± 8.984 pg/ml; P > 0.05 vs. HUVEC_lp_ + SP_Mreg_: 4.187 ± 4.187 pg/ml |

**Supplementary Tab. 3** Overview of non-significant results of Multiplex analysis of HUVEC_ep_ and HUVEC_lp_ cultivated with (+ SP_Mreg_) and without (+ Co) the addition of SP_Mreg_.
