# Supplementary material for: Secretory products from regulatory macrophages modulate senescence in human endothelial cells: implications for cardiovascular aging and diseases
Source: BMC Cardiovasc Disord. 2026 Mar 31;26:301. doi: 10.1186/s12872-026-05732-w (PMC13063746; doi:10.1186/s12872-026-05732-w)
Supplement: Supplementary file 3 — Supplementary Material 3. [file 12872_2026_5732_MOESM3_ESM.docx]

| HUVEC passage | HUVEC donor | SP_Mreg_ batches | Used in experiments |
| --- | --- | --- | --- |
| 4 | XIII | K325 | - cell size and volume - flow cytometric analysis of β-galactosidase, CD105 and ROS - intracellular ROS-levels - PCR analysis of CD105 |
| 4 | XIII | K327 | - cell size and volume - flow cytometric analysis of β-galactosidase, CD105 and ROS - intracellular ROS-levels - PCR analysis of CD105 |
| 5 | IX | K281 | - cell size and volume - flow cytometric analysis of β-galactosidase, CD105 and ROS - Multiplex ELISA of SASP associated factors |
| 5 | IX | K282 | - cell size and volume - flow cytometric analysis of β-galactosidase, CD105 and ROS - Multiplex ELISA of SASP associated factors |
| 5 | XIII | K326 | - cell size and volume - flow cytometric analysis of β-galactosidase, CD105 and ROS - intracellular ROS-levels - PCR analysis of CD105 |
| 6 | IX | K282 | - cell size and volume - flow cytometric analysis of β-galactosidase, CD105 and ROS - Multiplex ELISA of SASP associated factors |
| 6 | IX | K282 | - cell size and volume - flow cytometric analysis of β-galactosidase, CD105 and ROS |

**Supplementary Tab. 1** Overview of the origin and passage history of HUVEC_ep_, the preparation of SP_Mreg_ batches, and the specific HUVEC–SP_Mreg_ batch combinations applied in the experiments of this study.

| HUVEC passage | HUVEC donor | SP_Mreg_ batches | Used in experiment |
| --- | --- | --- | --- |
| 10 | IX | K281 | - cell size and volume - flow cytometric analysis of β-galactosidase, CD105 and ROS |
| 10 | XIII | K289 | - cell size and volume - flow cytometric analysis of β-galactosidase, CD105 and ROS - intracellular ROS-levels - PCR analysis of CD105 |
| 10 | XIII | K326 | - cell size and volume - flow cytometric analysis of β-galactosidase, CD105 and ROS - intracellular ROS-levels - PCR analysis of CD105 |
| 10 | XIII | K326 | - cell size and volume - flow cytometric analysis of β-galactosidase, CD105 and ROS - intracellular ROS-levels - PCR analysis of CD105 |
| 11 | IX | K277 | - cell size and volume - flow cytometric analysis of β-galactosidase, CD105 and ROS - Multiplex ELISA of SASP associated factors |
| 11 | IX | K281 | - cell size and volume - flow cytometric analysis of β-galactosidase, CD105 and ROS |
| 12 | IX | K282 | - cell size and volume - flow cytometric analysis of β-galactosidase, CD105 and ROS - Multiplex ELISA of SASP associated factors |
| 13 | IX | K279 | - cell size and volume - flow cytometric analysis of β-galactosidase, CD105 and ROS - Multiplex ELISA of SASP associated factors |

**Supplementary Tab. 2** Overview of the origin and passage history of HUVEC_lp_, the preparation of SP_Mreg_ batches, and the specific HUVEC–SP_Mreg_ batch combinations applied in the experiments of this study.
